# Does the ankle affect knee hyperextension during gait in hemiparetic stroke? A pilot study

**DOI:** 10.4102/sajp.v79i1.1926

**Published:** 2023-11-16

**Authors:** Catherine Cawood, Kholofelo Mashola

**Affiliations:** 1 Department of Physiotherapy, Faculty of Health Sciences, University of the Witwatersrand, Johannesburg, South Africa

**Keywords:** stroke, knee hyperextension, ankle, walking, muscle strength, tone, range of motion, stance phase

## Abstract

**Background:**

Knee hyperextension is common following stroke because of changes in joint range of motion (ROM), muscle tone and strength on the hemiparetic side. There is no clear consensus in the literature as to the cause of knee hyperextension during the stance phase of gait.

**Objectives:**

Our study aimed to determine the feasibility of methods to investigate the association between ankle joint function and knee hyperextension in patients with hemiparetic stroke during the stance phase of gait.

**Methods:**

We used a cross-sectional observational study to assess bilateral ankle muscle strength using a handheld dynamometer, ROM using a digital inclinometer and muscle tone using the Modified Tardieu Scale. The knee angles of the hemiparetic leg during the stance phase of gait were assessed using the Kinovea movement analysis software. Data were analysed using the Statistical Package for the Social Sciences with significance level set at 0.05 and 95% confidence intervals.

**Results:**

Twelve participants were included, and no alterations were necessary to the planned methodology. We found positive associations in six participants between the tibialis anterior muscle tone and the hemiparetic knee angles during heel strike, terminal stance and pre-swing phases (*p* < 0.05, *p* < 0.01 and *p* < 0.01, respectively).

**Conclusion:**

The results of the data analysis suggests that there may be a correlation between tibialis anterior muscle tone and knee hyperextension, a larger study will be imperative to confirm this association.

**Clinical implications:**

The methods described in our pilot study are feasible for a larger study to be conducted with the recommendations considered.

## Background

Restoration of walking is one of the main physiotherapy goals for many patients following a stroke, to be more functional in daily life and to allow for greater community participation (Cooper et al. 2012). Sixty percent to 80% of stroke survivors can walk independently by 6 months following a stroke (Veerbeek et al. 2011). However, stroke survivors are often left with functional limitations such as changes in motor control, joint range of motion (ROM), muscle tone, sensation and muscle strength (Li, Francisco & Zhou 2018; Lucareli & Greve 2008). These functional limitations result in reduced standing balance, increased energy expenditure, joint deformity, pain and muscle wasting (Cooper et al. 2012), which all then impact on the individual’s gait.

Gait comprises a full cycle that begins as one foot strikes the ground and ends when it strikes the ground again and is subdivided into stance and swing phases (Kharb et al. 2011; Silva & Stergiou 2020). The stance phase of gait can be described as the moment when the foot lands on the ground and includes the ‘weight acceptance’ phase and the ‘single limb support’ phase (Richardson et al. 2012). These two phases can be further classified into ‘initial contact’ and ‘loading response’ in the weight acceptance phase and ‘midstance’, ‘terminal stance’ and ‘pre-swing’ in the single limb support phase. Throughout the single limb support phase, the knee remains extended, although there is a small amount of knee flexion that occurs early in the stance phase (Balaban & Tok 2014). Individuals with stroke usually present with a shorter stance phase and increased swing phase on the hemiparetic side and knee hyperextension can occur during the heel strike or initial contact phase, loading response phase and midstance phase (Li et al. 2018; Richardson et al. 2012).

Knee hyperextension is described as extension of the hemiparetic knee past ‘anatomical zero’ and 40% – 60% of the stroke population present with knee hyperextension while walking (Cooper et al. 2012), although there is no clear consensus as to the cause of knee hyperextension. There is evidence that the ankle joint plays a role in knee hyperextension, with several factors causing altered ankle joint functioning that have an impact on the knee joint in patients with stroke during gait (An & Won 2016). Increased muscle tone in the ankle plantarflexor muscles causes early contraction of the ankle plantarflexors at ‘initial contact’, pulling the tibia posteriorly while the femur travels forward, causing knee hyperextension (Sheffler & Chae 2015). Spasticity in the ankle plantarflexors and shortening of the Achilles tendon may result in changes in ankle ROM, causing knee hyperextension at initial contact because of a lack of an antagonist muscle force (Higginson et al. 2006). Reduced ankle ROM during the stance phase of gait leads to the reduced ability to shift one’s centre of mass, leading to instability and balance deficits during gait, as well as soft tissue changes of the connective tissue and muscles of the ankle leading to biomechanical changes in gait (An & Won 2016). Maximal ankle dorsiflexion of 10° is achieved at midstance phase of gait and terminal stance phase (Ota et al. 2014). However, decreased dorsiflexion ROM hinders the forward translation of the tibia over the foot, which leads to compensation at the knee and altered spatiotemporal parameters such as decreased stride length, step length, cadence and walking speed (Ota et al. 2014).

Muscle weakness of the quadricep, hamstring and both ankle dorsiflexor and plantarflexor muscles of the hemiparetic lower limb has been shown to have an impact during the stance phase on knee hyperextension (An & Won 2016; Cooper et al. 2012; Sheffler & Chae 2015). Weakness of the quadricep muscle leads to reduced weight bearing ability (Cooper et al. 2012), while weakness of the hamstring muscle has an influence on knee hyperextension, especially if there is spasticity in the quadricep muscle (Springer et al. 2013). The gastrocnemius and hamstring muscles are thought to work in tandem during the stance phase of walking, to limit rapid knee hyperextension, but the exact action of the gastrocnemius and hamstring muscles in the stance phase of gait is not well demonstrated in the literature (Sheffler & Chae 2015; Springer et al. 2013). Cooper et al. (2012) suggest that tibialis anterior muscle weakness may also contribute to knee hyperextension during the stance phase of gait, as the ankle dorsiflexor muscles are active in the early stance phase to control foot descent to the floor and control the loading response.

During the stance phase of gait, knee hyperextension can allow individuals with stroke a certain amount of stability and provide a mechanism to control their affected lower limb, however, there is the potential for injury to the posterior capsular and ligamentous structures that may lead to pain, ligament laxity and bony deformity (Cooper et al. 2012; Richardson et al. 2012). Furthermore, knee hyperextension has the potential to negatively impact gait therapy, with a peak angle of 22° of knee hyperextension gait (particularly in the stance phase of gait). The goal for physiotherapy is to restore an efficient gait, where the patient can ambulate independently and safely. To do this, the causes of the patient’s altered gait pattern need to be identified and corrected to deliver the most effective and appropriate therapy. Our pilot study therefore aimed to determine the feasibility of the methods for a larger study to investigate the association between ankle joint function and knee hyperextension in patients with hemiparetic stroke during the stance phase of gait. We had three specific objectives:

- To assess and confirm the efficiency and ethical compliance of the proposed recruitment and consent procedures.
- To validate and refine inclusion and exclusion criteria.
- To test the appropriateness of instruments and feasibility of selected instruments for analysing:
  ■ the knee joint angles during the first three stance phases of gait (initial contact/heel strike, loading response and midstance)
  ■ the association between tibialis anterior and gastrocnemius muscle strength for ankle dorsiflexion and plantarflexion, respectively; muscle tone of the muscles of the ankle joint (tibialis anterior for ankle dorsiflexion and gastrocnemius for ankle plantarflexion) and joint ROM of ankle dorsiflexion and plantarflexion and hyperextension of the knee in the stance phase of gait.

## Methods

The methods potentially to be used by a larger study were used in our pilot study. A cross-sectional observational study with consecutive sampling was used where all consenting in- and out-patients at two rehabilitation facilities, which fitted the inclusion criteria, were approached to take part in our study. Potential participants were recruited between December 2020 and October 2021. The inclusion criteria included: unilateral hemiplegia from a single clinical stroke irrespective of the cause of stroke (including COVID-19 related causes), or the duration of the stroke; ability to walk independently (minimum of 10 m) either with or without an assistive device; no pathologies of the lower limb joints such as joint arthroplasty, fractures, osteoarthritis or rheumatoid arthritis and consenting adults (18 years and above).

Von Hoorhis and Morgan (2007) suggest that for regression equations with at least six predictors, a sample of a minimum of 10 participants per variable is appropriate. For a larger study, determining ankle involvement in knee hyperextension would include seven predictors namely: ankle muscle strength, muscle tone and ankle ROM of both agonists and antagonists, as well as the knee angle itself. Therefore, the sample size was calculated to be 7 × 10 participants per predictor variable that equates to 70 participants. For our pilot study, the rule of thumb was 10% of the sample size calculated for the larger study. However, when there is no previous information to base the sample size on, 12 participants were the recommended sample size as guided by Julious (2005).

## Data-collection procedure

Physiotherapists working at two rehabilitation facilities were asked to screen their patients for potential study participants, which included determining if their patients fitted the inclusion criteria. Ten visits were conducted to the two study sites, and no more than two potential participants were identified at any one time, meaning that a cluster of testing could not be performed in a single visit.

### Data-collection tools

A demographic information capture sheet was used to document data on the participant’s age, sex, months since stroke, affected side, type of assistive device used when walking, dominant side, height, weight and COVID-19-related questions (such as previous exposure and/or associated symptoms).

We used a handheld dynamometer as its use in isometric strength testing has been established in the stroke population (Andrews & Bohannon 2003). It is a convenient and appropriate method to test muscle strength with a high test-retest and inter-rater reliability (Mentiplay et al. 2015; Scott et al. 2004). Each participant was asked to perform three maximal isometric contractions for muscle groups on both the affected and unaffected sides being tested. The isometric contractions were held for 3 s, and the average of the maximal isometric contractions was used for data analysis. At no point did the strength of the participant’s affected and unaffected lower limbs exceed the first author’s own upper limb strength, to ensure reliability and validity of the strength measurements (Andrews & Bohannon 2003).

The Modified Tardieu Scale (MTS) to test spasticity was used to determine dynamic muscle tone (Abolhasani et al. 2012) and to differentiate between the peripheral (soft tissue changes) and the neural contributions (overactive stretch reflex) of spasticity (Singh et al. 2011). Furthermore, the MTS is more effective than the Modified Ashworth Scale (MAS) in identifying spasticity and differentiating it from contracture (Glinsky, 2016) and has good intra-rater reliability and validity (Banky et al. 2019; Singh et al. 2011). The MTS takes three measurements: R2, R1 and R2−R1 to measure spasticity. The R2 is measured at a slow velocity with the passive ROM measured, while the R1 is measured at a fast velocity with the angle of muscle reaction. A larger R2–R1 value indicates spasticity, while a smaller R2–R1 value indicates muscle contracture (Abolhasani et al. 2012). Lastly, the quality of muscle reaction is graded based on 0–4 score. The dynamic spasticity of a muscle is the relationship between R1 and R2 and is calculated by subtracting R1 from R2. Three measurements were taken on the affected side, and the average of these measurements was used for data analysis. The quality of muscle reaction was measured using a grading system (Fayazi et al. 2014).

We measured joint ROM using a digital inclinometer on a smartphone, which has been found to be a valid and reliable tool to measure joint ROM (intraclass correlation coefficient = 0.96–0.99) and is both easily accessible and inexpensive (Konor et al. 2012; Vohralik et al. 2015). The weight-bearing lunge test is more accurate when measuring ankle dorsiflexion ROM and is more reliable (intraclass correlation coefficient [ICC] = 0.93–0.96) than a non-weight-bearing position (ICC 0.32–0.72) (Bennell et al. 1998; Venturini et al. 2006). A weight-bearing position also reflects the available ROM during functional activities such as ambulation (Venturini et al. 2006). Three measures of the dorsiflexion lunge test and ankle plantarflexion ROM on both the affected and unaffected sides were recorded, and for data analysis, the average of these measures of both tests on each lower limb was used (Cox et al. 2018).

Analysis of movement uses tools to assess joint kinematics and changes in ROM and muscle activity during activities such as walking. Kinovea movement analysis software was used to analyse knee joint angles during gait. Kinovea is an open-access, 2D motion analysis software, which has been found to be an accurate and reliable instrument when measuring angular and distance data up to 5 m from the object and at an angle of 90° (Puig-Diví et al. 2017). Three-coloured stickers were placed on the participant’s paretic lower limb: over the greater trochanter, over the lateral femoral epicondyle and over the lateral malleolus as guided by Cooper et al. (2012). The participants were asked to then walk 10m at their own chosen speed. The walkway was demarcated with tape and the same walkway was used for all measures at each testing site. The participants walked barefoot and with their chosen assistive device. The camera was placed at a 90° angle less than 5 m away from the participants, at the level of their knee as they walked as in Puig-Diví et al. (2017). The video was imported into Kinovea movement analysis software and the ankle, knee and hip angles of the affected side were measured during the three different stance sub-phases.

All outcome measures were undertaken by the first author.

### Data analysis

Data were analysed using the Statistical Package for the Social Sciences (SPSS) and the significant level was set at *p* < 0.05 with a 95% confidence interval. Median and interquartile ranges were used to analyse descriptive statistics, while the independent Mann-Whitney test was used to compare differences between the affected and unaffected lower limbs. Spearman’s Rho correlation tests were used to analyse associations between the ankle components and knee hyperextension.

## Results

Our pilot study included 12 participants, 10 men (83.30%) and two women (16.70%). The youngest participant was 27 years old and the oldest 77. The median age of the participants was 46 (± 20) with the median months since stroke onset being 4.00 months (± 11.13). Most of the participants had left hemiplegia compared to right hemiplegia (*n* = 8, 66.7% vs *n* = 4, 33.3%) and all participants were right dominant. Half of the participants did not require an assistive device (50%), while two participants required a crutch for longer distances community or outdoor mobility (16.7%), and four participants required a quadrupod (33.3%) (Table 1).

**TABLE 1:** Age, height, weight and months since stroke normal distribution data (*n* = 12).

Demographic data	Age in years	Height (cm)	Weight (kg)	Months since stroke
Median	46.00	172.50	79.00	4.00
Interquartile range	27	12	20	11.13
Minimum	27	157	57	0.25
Maximum	77	196	165	13.00

## Description of the biomechanics of the ankle complex

### Muscle strength, tone and range of movement

Muscle strength data of the unaffected and affected dorsiflexors and plantar flexors are depicted in Table 2 and are normally distributed except plantarflexion muscle strength of the unaffected ankle.

**TABLE 2:** Muscle strength, tone and range of movement of the ankle distribution data (*n* = 12).

Ankle	Min	Max	Mean	Median	Interquartile range	Skewness		Kurtosis	
						Statistic	Std. error	Statistic	Std. error
**Muscle strength**									
Dorsiflexion (unaffected)	4.00	20.00	11.67	12.67	4.49	0.12	0.64	0.75	1.23
Plantarflexion (unaffected)	4.83	26.67	12.93	12.50	3.42	1.51	0.64	4.73	1.23
Dorsiflexion (affected)	0.00	10.00	6.08	6.67	4.08	−0.75	0.64	0.53	1.23
Plantarflexion (affected)	0.00	17.00	8.39	8.34	1.59	0.14	0.64	3.59	1.23
**Muscle tone**									
R2 Dorsiflexion – Passive ROM	−15.67	12.33	1.028	0.84	13.67	−0.55	0.64	−0.39	1.23
R1 Dorsiflexion – ROM with rapid stretch	−16.67	10.00	−6.25	−5.17	13.00	0.58	0.64	−0.32	1.23
R2-R1 Dorsiflexion	−0.67	22.33	7.17	6.33	9.83	0.89	0.64	0.33	1.23
R2 Plantarflexion – Passive ROM	35.00	50.00	42.64	42.17	9.91	0.14	0.64	−1.46	1.23
R1 Plantarflexion – ROM with rapid stretch	34.67	50.00	41.59	41.34	10.83	0.21	0.64	−1.76	1.23
R2-R1 Plantarflexion	−1.00	4.00	0.97	0.33	2.08	0.83	0.64	0.11	1.23
**Range of movement**									
ROM dorsiflexion (unaffected)	20.53	36.80	29.62	30.18	10.45	−0.50	0.64	−0.96	1.23
ROM plantarflexion (unaffected)	24.23	45.07	33.97	33.28	11.49	0.18	0.64	−0.91	1.23
ROM dorsiflexion (affected)	10.53	35.23	24.99	25.39	8.49	−0.51	0.64	0.28	1.23
ROM plantarflexion (affected)	21.27	42.60	31.73	31.64	13.87	0.01	0.64	−1.83	1.23

Min, minimum; Max, maximum; Std. error, standard error; ROM, range of motion.

Table 2 depicts a significant difference between dorsiflexion muscle strength (F = 1.26; *p* = 0.27; Cohen’s d = 3.56) and plantarflexion muscle strength (F = 0.38; *p* = 0.55; Cohen’s d = 4.57) of the affected ankle as compared to the unaffected ankle, with the muscle strength of the unaffected side showing higher medians than the affected side. Our finding is consistent with the hemiparetic clinical presentation.

Slow velocity passive muscle tone of the affected gastrocnemius had a median of 0.84 (± 13.67), and a rapid velocity stretch had a median of –5.17 (± 13), with the R2-R1 dynamic muscle tone having a median of 6.33 (± 9.83). Slow velocity passive muscle tone of the tibialis anterior measured had a median of 42.17 (± 9.91), and a rapid velocity stretch had a median of 41.33 (± 10.83), with the R2-R1 dynamic muscle tone median being 0.33 (± 2.08). The data of muscle tone of the ankle are normally distributed except the dynamic muscle tone of the tibialis anterior muscle (R2-R1) (Table 2). The quality of the muscle reaction was also measured for the affected tibialis anterior and gastrocnemius muscles. The gastrocnemius had more varied muscle reaction qualities ranging from 0 to 4, while the tibialis anterior was only given a 0 or 1 rating (Table 3).

**TABLE 3:** Quality of muscle reaction for tibialis anterior and gastrocnemius muscle tone (*n* = 12).

Quality of muscle reaction		Gastrocnemius		Tibialis anterior	
		*n*	%	*n*	%
0	No resistance throughout the course of the passive movement	3	25.0	6	50
1	Slight resistance through the course of passive movement; no clear ‘catch’ at a precise angle	1	8.3	6	50
2	Clear catch at a precise angle, interrupting the passive movement, followed by release	7	58.3	0	0
3	Fatigable clonus (10 s when maintaining the pressure) appearing at a precise angle	0	0.0	0	0
4	Un-fatigable clonus (more than 10 s when maintaining the pressure) at a precise angle	1	8.3	0	0
5	Joint is immovable	0	0.0	0	0

Joint range of movement (ROM) of the unaffected dorsiflexion had a median of 30.18 (± 10.45), and its affected counterpart had a median of 25.39 (± 8.49). The unaffected plantarflexion ROM had a median of 33.28 (± 11.49), while the affected plantarflexion had a median of 31.64 (± 13.87). The data of ROM of the ankle are normally distributed (Table 2). There were no significant differences between plantarflexion ROM (F = 2.15; *p* = 0.16) and dorsiflexion ROM of the affected versus unaffected ankles (F = 0.43; *p* = 0.52).

### Gait analysis: Changes in knee angles during stance phase

The angle of the knee of the hemiparetic side was measured at each phase of stance phase during gait. Three of the participants presented with knee hyperextension during stance phase of gait and the median angles are depicted in Table 4. The data of the knee angles measured are normally distributed with the knee angles showing greater knee flexion during stance phase, according to the norms (Table 4).

**TABLE 4:** Hemiparetic knee angles during the stance phases of gait distribution data (*n* = 12).

Stance phases during gait	Minimum	Maximum	Median	Interquartile range	Skewness		Kurtosis	
					Statistic	Std. error	Statistic	Std. error
Initial contact	−1.6	21.3	12.2	9.4	−1.2	0.64	−0.49	1.23
Loading response	−3.3	29.9	15.0	15.7	−0.17	0.64	−0.51	1.23
Midstance	−11.1	25.7	14.6	18.2	−0.74	0.64	−0.04	1.23
Terminal stance	−7.4	24.3	13.2	20.8	−0.47	0.64	−1.2	1.23
Pre-swing	11.3	24.0	19.5	8.3	−0.67	0.64	−0.76	1.23

Std. error, standard error.

### Association during gait stance phase between the ankle components assessed and knee hyperextension

The correlation tests between decreased tibialis anterior muscle tone and knee angles (increased knee flexion) showed a strong and positive relationship to the hemiparetic knee angles during the stance phase of gait (r_12_ = 0.662; *p* = 0.019). Upon further investigation, we found significant relationships between tibialis anterior muscle tone and knee angles during all stages of the stance phase of gait, except the loading response and midstance (Table 5). Gastrocnemius muscle tone was not significantly related to the knee angles during any of the stages of the stance phase – initial contact (*p* = 0.22), loading response (*p* = 0.45), midstance (*p* = 0.24), terminal (*p* = 0.48) or pre-swing (*p* = 0.85). Table 5 also depicts that the affected ankle’s muscle strength and ROM were not significantly related to the knee angles at the stance phase of gait.

**TABLE 5:** Correlation data between ankle muscle strength, ankle range of motion and hemiparetic knee angles during the stance phases of gait (*n* = 12).

Stance phasesduring gait	Muscle strength		Range of movement		Muscle tone	
	Dorsi-flexion	Plantar-flexion	Dorsi-flexion	Plantar-flexion	Dorsi-flexion	Plantar-flexion
**Initial contact**						
Spearman’s rho	−0.382	0.127	−0.098	−0.343	0.098	0.542
*p*	0.220	0.694	0.762	0.276	0.761	0.069
**Loading response**						
Spearman’s rho	0.239	0.226	−0.119	0.056	0.109	0.321
*p*	0.455	0.480	0.713	0.863	0.736	0.309
**Midstance**						
Spearman’s rho	0.368	0.212	−0.399	0.217	0.302	0.492
*p*	0.239	0.508	0.199	0.499	0.340	0.104
**Terminal stance**						
Spearman’s rho	0.225	0.226	−0.657*	0.098	0.496	0.745**
*p*	0.483	0.480	0.020	0.762	0.101	0.005
**Pre-swing**						
Spearman’s rho	0.062	0.451	−0.508	0.119	0.308	0.672*
*p*	0.849	0.141	0.092	0.712	0.330	0.017

* , Correlation is significant at the 0.05 level (2-tailed);

** , Correlation is significant at the 0.01 level (2-tailed).

A multiple regression analysis revealed that dorsiflexion and plantarflexion muscle strength; dorsiflexion and plantarflexion ROM; as well as tibialis anterior and gastrocnemius muscle tone did not impact the knee angles (Table 6).

**TABLE 6:** Multiple regression and tests between subject-effects (*n* = 12).

Ankle	Variance (%)	F	*df*	*p*
Muscle strength DF	38.2	0.74	5.6	0.621
Muscle strength PF	29.9	0.51	5.6	0.763
ROM DF	18.3	0.27	5.6	0.921
ROM PF	53.0	1.35	5.6	0.362
Muscle tone DF	46.1	1.03	5.6	0.484
Muscle tone PF	71.9	3.07	5.6	0.100

ROM, range of motion; df, degree of freedom; PF, plantarflexion; DF, dorsiflexion.

## Feasibility of our study

### Recruitment and consent procedures

Five facilities were approached and two granted permissions to conduct our study on their premises with their in- and out-patients. There were 15 potential participants who were approached and three were excluded because of multiple strokes (*n* = 1), lower limb pathology (*n* = 1) and unable to understand and sign informed consent (*n* = 1). The recruitment of the 12 pilot study participants took 10 months to complete, from December 2020 to October 2021, which was during the COVID-19 pandemic.

### Data-collection tools

The total time taken to complete the assessment was approximately 20 min. The demographic information capture sheet took approximately 5 min to complete, while the handheld dynamometry to assess muscle strength took less than 1 min to complete. Testing spasticity using the Modified Tardieu Scale took approximately 5 min. Ankle dorsiflexion was measured with an inclinometer while the participant performed a weightbearing lunge test against a wall, while ankle plantarflexion ROM was measured in a supine position. Three measures of the dorsiflexion lunge test and ankle plantarflexion ROM on both the affected and unaffected sides were recorded. This test took approximately 5 min to test. The participants were asked to then walk 10 m at their own chosen speed that took approximately 2 min to complete.

## Discussion

### Appropriateness of instruments

The data-collection tools were all convenient, quick, easy-to-use and can be used in a larger study to achieve the same objectives. For better reliability and accuracy, the camera could be placed on a tripod with wheels so that it is kept at the same angle and distance from the participant’s knee for the entire walk. Van der Kruk and Reijne (2018) suggest that the use of reflective markers as used in optoelectronic systems such as Vicon is more accurate and could be considered in place of plain coloured stickers. The knee angle norms were compared to accepted norms as per the normal range and zero line; however, the larger study should consider not only analysing each patient but also compare their affected with their unaffected side.

### Demographic data

We only included individuals who were post-stroke who could walk independently, and the median months since stroke onset was 4.0 months and Veerbeek et al. (2011) found that 60% – 80% of stroke survivors can walk independently by 6 months following a stroke (Veerbeek et al. 2011). All the participants were right-hand dominant while the hemiparetic side of the participants was distributed with most of the participants having left-sided hemiplegia. This distribution is not what has been seen in the literature as several studies have shown that left-sided strokes (right-sided hemiplegia) are more frequent than right-sided strokes (left-sided hemiplegia) (Foerch et al. 2005; Hedna et al. 2013). Right-sided strokes may result in problems with visuo-spatial perception, which would affect balance and mobility especially in more complex environments (John Hopkins Medicine 2021) and thus play a role in gait patterns.

### Muscle strength

We found significant weakness of both gastrocnemius and tibialis anterior muscles when compared to the unaffected side. Muscle weakness of the gastrocnemius and tibialis anterior muscles has been shown to be linked to knee hyperextension during the stance phase (An & Won 2016; Cooper et al. 2012; Sheffler & Chae 2015). Cooper et al. (2012) suggest that tibialis anterior is active in the early stance phase of gait to control foot descent to the floor and control the loading response, and weakness of this muscle during these phases may contribute to knee hyperextension. The gastrocnemius muscle is thought to prevent rapid knee hyperextension, but the exact action of it in the stance phase of gait is not well-demonstrated (Sheffler & Chae 2015; Springer et al. 2013). While our study showed no significant relationship between muscle strength and the hemiparetic knee angles during the stance phase of gait, muscle strength of the ankle showed a 38.2% variance for dorsiflexion muscle strength and 29.9% variance for plantarflexion muscle strength of the hemiparetic knee angles. As most of the participants presented with increased knee flexion during stance phase, this may show a greater link, during stance phase, between tibialis anterior muscle weakness and increased knee flexion, which is contradictory to Cooper et al. (2012). This warrants further investigation using a larger sample size.

### Muscle tone

We found a greater dynamic component of spasticity, as well as phasic tone in the gastrocnemius of the affected lower limb compared to the tibialis anterior. This was also apparent with the subjective rating of the quality of the muscle reaction. The gastrocnemius had more varied muscle reaction qualities ranging from 0 to 4 with most having a rating of 2 (58.33%), while the tibialis anterior was only given a 0 or 1 rating. Fayazi et al. (2014) found that all their 30 participants rated either a muscle reaction of 2 (33.33%) or 3 (66.67%). We found tibialis anterior muscle tone had a strong and positive relationship to the hemiparetic knee angles during the stance phase of gait. Increased muscle tone in the gastrocnemius muscle causes early contraction of the ankle plantar flexors at ‘initial contact’, pulling the tibia posteriorly while the femur travels forward, causing knee hyperextension (Balaban & Tok 2014; Sheffler & Chae 2015). This was not the case in our study, as there was no significant relationship between gastrocnemius muscle tone and knee hyperextension; however, only three participants presented with knee hyperextension and thus a conclusion cannot be drawn. During the stance phase, most of our participants presented with increased knee flexion, and the increased gastrocnemius muscle tone may still play a greater role in knee hyperextension than tibialis anterior muscle tone, similar to Balaban and Tok (2014), Sheffler and Chae (2015), Higginson et al. (2006). Decreased tibialis muscle tone was evident in participants who presented with increased knee flexion during the stance phase and therefore seems to not play a role in knee hyperextension.

### Ankle joint range of motion

Although ankle ROM of the affected dorsiflexion and plantarflexion were less than the unaffected side, we did not find any significant differences between the ankle ROM between the affected and unaffected sides. Nevertheless, decreased ankle ROM during the stance phase of gait leads to the reduced ability to shift one’s centre of mass, leading to instability and balance deficits during gait as well as soft tissue changes of the connective tissue and muscles of the ankle leading to biomechanical changes in gait (An & Won 2016). The greater difference in ankle dorsiflexion ROM between the affected and unaffected lower limbs we observed ties in with the increased muscle tone of the gastrocnemius. Spasticity in the ankle plantar flexors results in changes in ankle ROM, causing knee hyperextension at initial contact because of a lack of an antagonist muscle force (Higginson et al. 2006). While we showed no significant relationship between ankle ROM and the hemiparetic knee angles during the stance phase of gait, ankle ROM of the ankle showed 53% variance for plantarflexion ROM, and 18.3% variance for dorsiflexion ROM on hemiparetic knee angles. As most of the participants presented with increased flexion of the knee rather than knee hyperextension during the stance phase, it may show a greater link with plantarflexion ROM and increased knee flexion.

### Knee angles during the stance phase of gait

In hemiparetic gait, knee hyperextension can occur during the heel strike or initial contact phase, loading response phase and midstance phase (Richardson et al. 2012). Interestingly, we only had one participant presenting with ‘true’ knee hyperextension (less than anatomical zero) during gait. Although not ‘true’ knee hyperextension, five participants presented with knee angles less than the norm during loading response (knee was more extended), and three participants had knee hyperextension during the terminal stance phase. Regardless of the hyperextension being true or not, 40% – 60% of the stroke population present with knee hyperextension while walking (Cooper et al. 2012), which is like our findings. There are three distinctive knee patterns during the stance phase in patients with stroke, the first being increased flexion of the knee at initial contact, which is more commonly seen in the early post-stroke period and is associated with poor quadriceps activation (Balaban & Tok 2014; Sheffler & Chae 2015). The second distinctive knee pattern is decreased knee flexion early on, followed by knee hyperextension in the later stance phases. The third knee pattern seen is excessive knee hyperextension throughout the entire stance phase (seen in more chronic patients with stroke) (Balaban & Tok 2014; Sheffler & Chae 2015). Most of our participants presented with increased flexion of the knee in all the stance phases, with decreased flexion of the knee only in pre-swing. During the pre-swing phase, all the participants had a more extended knee than the norm of 35° – 40°. This ties in with patients with early post stroke having increased flexion of the knee early on in the stance phase (Balaban & Tok 2014; Sheffler & Chae 2015).

### Association between the ankle components assessed and knee hyperextension

Despite finding associations with dorsiflexion ROM and dorsiflexion muscle strength, both gastrocnemius and tibialis anterior muscle strength of the affected lower limb were not associated with the knee angles at initial contact, loading response, midstance, terminal, or pre-swing. During the stance phase, most of the participants presented with increased knee flexion, and this may show a greater link between tibialis anterior muscle weakness and increased knee flexion. This is, however, contradictory to Cooper et al. (2012), who suggest that tibialis anterior is active in the early stance phase of gait to control foot descent to the floor and control the loading response, and weakness of this muscle during these phases may contribute to knee hyperextension.

Plantarflexion and dorsiflexion ROM were also not associated with the knee angles at initial contact, loading response, midstance, terminal or pre-swing. This may be because of the small sample size, or because passive ROM was measured, and active ROM plays a greater role during walking. We found dorsiflexion ROM and dorsiflexion muscle strength to be associated, which could suggest that if the tibialis anterior is strong enough, it may counteract the tone in the gastrocnemius and reduce the changes to ankle ROM into dorsiflexion. However, gastrocnemius spasticity causes reduced ankle dorsiflexion ROM as well as reduced plantarflexion muscle strength (Aggarwal, Walia & Noohu 2013; Ng & Hui-Chan 2012), which is unsurprising as the gastrocnemius muscle is greater in size and can thus easily overpower the tibialis anterior muscle actions.

Increased muscle tone in gastrocnemius causes early contraction of the ankle plantar flexors at ‘initial contact’, pulling the tibia posteriorly while the femur travels forward, causing knee hyperextension (Balaban & Tok 2014; Sheffler & Chae 2015). However, this is not what we found, as increased tibialis anterior muscle tone had a greater impact on the knee angles than gastrocnemius. Most of our participants presented with increased knee flexion during stance phase, and so increased gastrocnemius muscle tone may still play a greater role in knee hyperextension than tibialis anterior muscle tone, which is what we see in a clinical setting.

### Limitations of our study and recommendation for future research

As this is a pilot study, we recommend that these results be considered with caution and not be generalised to the whole stroke population as we conducted our study to precede a larger study. The sample size may not have had a great enough statistical power to reduce a Type-II error. For a larger study, it is advised that a large catchment area is included with both private and public healthcare facilities. Additional information could be added to the socio-demographic capture sheet to gain a better clinical presentation of the participant: type of stroke, cause of stroke and co-morbidities could be established to gain the full clinical features of the participants. Balance, mobility status and the functional level of the participant should be measured with appropriate outcome measures.

We have shown the feasibility of the methodology described for a larger study to be conducted, and we recommend that this be conducted with the modifications stated above with a larger sample size. Because of our study being aimed to inform the methods for a larger study, we do not recommend these findings for clinical practice.

## Conclusion

Our pilot study’s aim was to determine if the methods used are feasible for a larger study to determine the association between ankle muscle strength, muscle tone, joint ROM and knee hyperextension in hemiparetic stroke patients with hemiparetic stroke during gait stance phase. Because of the restrictions of the COVID-19 pandemic, permission to conduct our study was restricted at some healthcare facilities, which reduced the study pool. The outcome measures selected were easy to use and not time-consuming to administer, and the entire assessment took approximately 20 min to complete. The results of the data analysis suggest that there may be a correlation between tibialis anterior muscle tone and knee hyperextension, a larger study will be important to confirm this association.
